# Discordance between self-report and performance-based outcomes: Contribution of psychosocial factors

**DOI:** 10.1177/13591053241253895

**Published:** 2024-05-27

**Authors:** Helen Razmjou, Susan Robarts, Suzanne Denis, Amy Wainwright, Patricia Dickson, John Murnaghan

**Affiliations:** 1 Sunnybrook Health Sciences Centre, Canada; 2 University of Toronto, Canada

**Keywords:** disability, performance-based outcomes, response shift, self-report

## Abstract

The purpose of this study was to examine the role of psychosocial factors in the discordance between perceived and observed physical disability in patients with osteoarthritis of the hip or knee joint. This was a cross-sectional study of patients seen for consideration of joint arthroplasty surgery. Patients completed a psychosocial outcome measure, a patient self-reported functional scale, and two performance-based tests. Data of 121 patients, mean age, 67 (8), 81 (67%) females were used for analysis. The fear avoidance and positive affect domains had the strongest association with the discordance between the self-report and both performance outcome measures. Age, gender, and severity of osteoarthritis were associated with discordance in relation to walking. Fear avoidance beliefs and positive affect play important roles in perception of pain and function. Age, gender, and severity of arthritis should be taken into consideration for a more holistic approach to arthritis care.

## Introduction

The impact of psychosocial factors on physical function was first acknowledged by Roy Grinker in 1952 (Grinker, 1994) and expanded upon by George Engel in 1977 (Engel, 1977). Today, it is well established that biomedical models are unable to solely explain the discrepancy between disease severity and perceived disability (Engel, 1977; Epstein, 2014; Papadimitriou, 2017). A biopsychosocial model incorporates all elements of well-being (biological, psychosocial, and social) and acknowledges the role of personality, beliefs, affect, and the surrounding environment of a person in manifestation of physical disability. Indeed, it is through understanding the mind-body connection that one could provide effective care to a person suffering from chronic and disabling conditions.

Osteoarthritis (OA) of the hip and knee is a progressive condition and remains one of the leading causes of disability in older adults. Despite the significant economic burden of this condition on health care costs (Chen et al., 2023; Swain et al., 2023), there is limited information on the importance of affect, coping, and resilience on perception of difficulty in performing daily tasks in patients with OA of the hip or knee joint (Dayton et al., 2016; Lentz et al., 2020; Pua et al., 2023). The role of general psychological distress, such as anxiety and depression, and pain-related distress manifesting as catastrophic thinking about arthritis pain and reduced coping and self-efficacy for managing pain has been highlighted by Lentz et al. (2020). The authors concluded that management of severe OA through biomedical interventions alone (e.g. corticosteroid injection or joint replacement), which only address the joint pathology will not fully meet the needs of many patients with OA due to their concomitant psychological distress.

Traditionally, disability measurement has been conducted in two ways: Patient-reported outcome measures (PROMs) and performance-based tests. PROMs rely on patient self-report and measure the degree of perceived difficulty or inability in performing certain activities. PROMs have been utilized in the assessment and management of OA since the 1980s and are considered inexpensive and convenient for the patient. However, they are highly subjective to the perception of “difficulty” or “inability” and rely on the patient’s experience, recall, emotional state, and the patient’s environment. In addition, PROMs may be difficult to complete by elderly patients, those who are not fluent in the tool’s language, or those with cognitive difficulties (Coman and Richardson, 2006; Guralnik et al., 1989; Kempen et al., 1996; Myers et al., 1993; Reuben et al., 2004).

Performance measures involve the actual assessment of the person’s abilities in performing isolated tasks at a single time point, representing the impact of the disease at that specific point in time (Fors et al., 2006). Compared with self-report outcomes, performance measures are less affected by the mental state of the patient, as they represent the actual ability rather than presumed capability of a person. They can however be time consuming and require trained personnel and adequate space for testing. Additionally, they may be inconvenient, as they require the patient to be present in the clinic or hospital. Moreover, they are subject to observer bias, instruction bias, and motivation of the patient to perform the test (Dayton et al., 2016; Myers et al., 1993).

There is limited literature on the discordance between actual and perceived physical function in the field of orthopedics. Some investigators have highlighted the role of psychosocial factors in the observed discrepancy between self-report and performance-based outcomes (Kok et al., 2020; Lentz et al., 2020; Pua et al., 2023; Twardzik et al., 2024; Wilfong et al., 2020). Lack of concordance between self-report and performance-based measures presents an opportunity to improve the management of chronic complex illnesses that may be optimized by addressing the impeding non-physical factors and the existing gap in the management of OA. In addition, it is important to examine which components of psychosocial well-being (e.g. positive affect vs negative affect) are more important in the perception of pain and disability as they represent different traits and mental states and have different management strategies. Thus, further assessment of psychological well-being in perception of disability warrants further examination.

The purpose of this study was to examine the role of psychosocial factors in the discordance between perceived and observed physical disability in patients with OA of the hip or knee joint, while accounting for severity of arthritis, age, and gender. We hypothesized that psychosocial factors play a vital role in explaining the difference between self-report and performance-based measures. More specifically, patients who overestimate their self-report physical function were expected to have a more positive appraisal of life (e.g. better positive affect, higher resilience, better coping, and less fear-avoidance beliefs and catastrophic thinking). The role of OA severity, age, and gender was explored.

## Materials and methods

This was a cross-sectional study of patients with knee or hip OA who were seen at a tertiary care center for consideration of joint arthroplasty. The study protocol was approved by the Human Research Ethics Board of the Sunnybrook Health Sciences Centre and patients provided informed consent for participation in the study.

### Clinical examination

Clinical examination was conducted by advanced practice providers (APPs) who were physiotherapists or occupational therapists with advanced formal education and special training and included a comprehensive musculoskeletal examination. The APPs completed a standardized assessment tool used in the Rapid Access Clinics; the osteoarthritis severity scoring system to determine appropriateness for surgical consultation. The severity score is based on three distinct components: clinical (history, pain intensity, physical examination), functional (scores from self-report and performance measures), and radiological examination (severity of arthritic changes on plain radiographs). The total severity score is calculated by summing all three scores, ranging between 0 and 8 with higher numbers indicating a higher severity. The severity score has shown validity in the OA population (Robarts et al., 2023a, 2021).

### Patient-reported outcome measures (PROMS)

The Lower Extremity Functional Scale (LEFS; Binkley et al., 1999) and P4 (4-item pain intensity measure; Spadoni et al., 2004) were used as PROMS in the present study. The LEFS has 20 questions with four responses for each question and the total score ranges from 0 to 80 with the highest numbers indicating better function. The items of the P4 (0–40) address pain in the morning, afternoon, evening, and with activity. Each item is scored on an 11-point numeric pain scale, with higher numbers indicating higher pain. LEFS and P4 are reported to have sufficient levels of reliability and validity in patients with lower extremity arthritis (Binkley et al., 1999; Kennedy et al., 2008; Mehta et al., 2016; Spadoni et al., 2004).

### Performance-based measures

Patients completed two performance-based tests with an experienced clinician: the 40-m fast-paced walk test (Dobson et al., 2013) and the 30-second Chair Stand Test (CST; Jones et al., 1999). The 40-m fast-paced walk test is recorded in seconds and involves walking a 10 m distance, turning around at a cone, returning and repeating for a total distance of 40 m, as quickly and safely as possible. The CST documents the maximum number of sit-stand repetitions possible in a 30 second period. These performance measures are reported to be valid and reliable in patients with hip/knee arthritis and are good indicators of lower extremity muscle strength (Dobson et al., 2013; Jones et al., 1999; Master et al., 2021). To avoid temporal discrepancy between self-reported and performance-based measures, both outcomes were captured at the same time as part of the clinical assessment.

### Psychosocial assessment

All patients completed the 10-item Optimal Screening for Prediction of Referral and Outcome Yellow Flags (OSPRO-YF) Assessment Tool, which examines both general and pain-related psychological distress constructs of depression, anxiety, fear of movement, and self-efficacy for managing one’s own pain (Lentz et al., 2016). This questionnaire has three domains: negative mood, fear avoidance, and positive affect domains. The negative mood (anxiety, depression, anger symptoms) and fear avoidance (pain catastrophizing, activity and occupation-related fear) address pain vulnerability. The positive affect domain (self-efficacy for pain and rehabilitation, and acceptance for chronic pain) documents resilience and coping (Lentz et al., 2020). The total score of each construct is in a continuous metric and is calculated by summing all item responses with the reverse score for pain resilience items. Higher scores of each domain and the total score indicate higher psychological distress and pain vulnerability and lower pain resilience. The OSPRO-YF survey has shown validity and reliability in patients with a variety of musculoskeletal pathologies (George et al., 2017, 2020; Razmjou et al., 2021; Robarts et al., 2023b) and specifically, in patients with OA of the hip and knee joints (Lentz et al., 2020).

### Statistical analysis

Descriptive analyses of relevant patient characteristics and outcomes (LEFS, P4, performance measures, osteoarthritis severity score, and OSPRO-YF domains) were performed. The scores of the LEFS, OSPRO-YF, CST, and 40-m fast-paced walk were rescaled to a 0–100 scale to equate the scales and facilitate comparability between different scales (Wilfong et al., 2020). The range for the LEFS and OSPRO-YF varies between 0 and 80 and 3 and 53 respectively. The range of 40-m fast-paced walk scale in our sample was 0–68, therefore the formula used to rescale was (40-m fast-paced walk–0)/(68–0) × 100. The range of the CST score in our sample was 0–28 repetitions, and the formula used to rescale was (CST–0)/(28–0) × 100.

Discordance referred to the difference between the self-assessed level of physical functioning and the actual level of performance on physical tests. The discordance score was calculated by subtracting the rescaled LEFS score from the rescaled 40-m fast-paced walk and the CST score with zero indicating no difference between the LEFS and the performance-based measures. Positive discordance scores resulted if an individual had a higher (i.e. better) self-reported score than their performance-based score (overestimation). A negative discordance score resulted if an individual had a lower (i.e. worse) self-reported score than their performance-based score (underestimation). Generalized linear models (GLM) examined the association between the discordance (dependent variable) and each domain of OSPRO-YF (negative mood, fear avoidance, and positive affect), accounting for age (continuous data), gender (male/female), and OA severity. In this manuscript, the binomial factor of male/female incorporated both sex and gender characteristics. Plausible interactions were examined.

## Results

One hundred and twenty-one patients, mean age: 67 (8), 81 (67%) females: 40 (33%) males: 79 (65%) knees, and 42 (35%) hips were included in the study. Table 1 shows the demographic and characteristics of the sample.

**Table 1.** Characteristics of the sample included (*N* = 121).

Variables	Number (percentage/SD)
Age: Mean (SD)	67 (8), min 47, max 82
Sex: *n* (%)	
Female	81 (67)
Male	40 (33)
Affected joint: *n* (%)	
Knee	79 (65)
Hip	42 (35)
Walking devices: *n* (%)	
Yes	28 (23)
No	93 (77)
Severity score: mean (SD)	
Clinical (0–3)	1.74 (0.54)
Functional (0–3)	1.72 (0.55)
Radiological (0–2)	1.73 (0.42)
Total score (0–8)	5.20 (1.13)
Outcome measures	
LEFS (0–80)	38 (15)
P4 (0–40)	21 (9)
Chair Sit Test (repetition)	11 (4)
40 m fast paced walk (seconds)	33 (9)
OSPRO-YF domains	
Negative affect (2–11)	3.88 (2)
Fear avoidance (1–20)	10.81 (4)
Positive affect (0–22)	13.09 (4)

LEFS: Lower Extremity Functional Score; P4: 4-item pain intensity measure; OSPRO-YF: Optimal Screening for Prediction of Referral and Outcome Yellow Flags.

**Figure 1.**
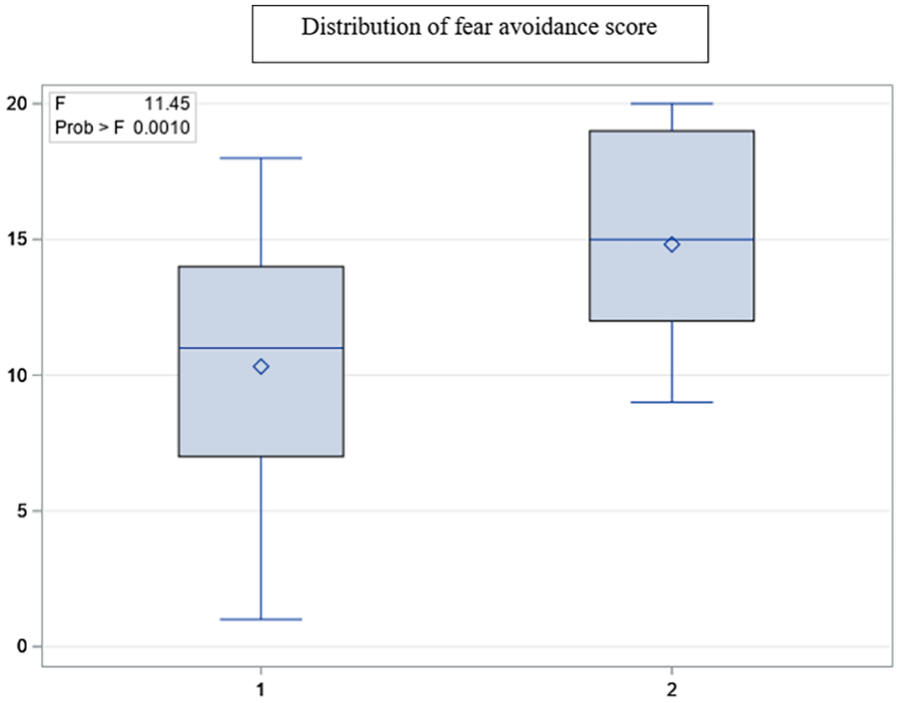
Group differences in fear avoidance score. Group1: Overestimated self-report functional abilities in relation to actual walking ability. Group1: Underestimated self-report functional abilities in relation to actual walking ability.

Patients who overestimated their function, reported less fear avoidance, *F*_(1, 119)_ = 11.45, *p* = 0.001 (Figure 1) and better positive affect, *F*_(1, 119)_ = 9.56, *p* = 0.0003 in relation to their actual walking time and less fear avoidance, *F* = 6.61 _(1, 119)_, *p* = 0.01, and better positive affect *F*_(1, 119)_ = 7.57, *p* = 0.007, with respect to their ability to sit-stand from a chair. Overall, negative affect did not play an important role in discordance between LEFS and walking test or the number of sit-stands from a chair.

Assessment of the relationship between discordance and domains of the OSPRO-YF (negative mood, fear avoidance, and positive affect) showed that the fear avoidance and the positive affect domains had the strongest association with the discordance between the LEFS and both performance measures (Table 2).

**Table 2.** Associations between predictors and discordance.

Predictors	Statistics (*F*_(4, 116)_, 95% CI, *p* value, ES*)
*LEFS and 40-m fast-paced walk discordance*	
Negative affect	*F* = 6.22 (0.22–20.1), *p* = 0.01, 5.11
Arthritis severity	*F* = 6.56 (0.30–20.7), *p* = 0.01, 5.35
Age	*F* = 5.56 (0.09–18.9), *p* = 0.011, 4.43
Gender	*F* = 2.98 (0.00–13.7), *p* = 0.08
Fear avoidance	*F* = 56.62 (28.32–94.1), *p* < 0.0001, 54.62
Arthritis severity	*F* = 7.33 (0.49–22.0), *p* = 0.008, 6.20
Age	*F* = 11.08 (1.72–28.4), *p* = 0.001, 9.89
Gender	*F* = 4.03 (0.10–15.8), *p* = 0.047, 2.96
Positive affects	*F* = 29.90 (11.37–56.9), *p* < 0.0001, 28.37
Arthritis severity	*F* = 7.42 (0.52–22.2), *p* = 0.008, 6.29
Age	*F* = 6.80 (0.36–21.1), *p* = 0.010, 5.69
Gender	*F* = 6.80 (0.36–21.1), *p* = 0.010, 5.68
*LEFS and 30-second Chair Stand Test discordance*	
Negative affect	*F* = 0.12 (0.00–4.93), *p* = 0.73
Arthritis severity	*F* = 1.43 (0.00–9.99), *p* = 0.23
Age	*F* = 0.15 (0.00–5.19), *p* = 0.70
Gender	*F* = 1.62 (0.00–10.48), *p* = 0.21
Fear avoidance	*F* = 23.36 (7.63–47.38), *p* < 0.0001, 21.93
Arthritis severity	*F* = 1.27 (0.00–9.54), *p* = 0.26
Age	*F* = 0.70 (0.00–7.82), *p* = 0.40
Gender	*F* = 2.71 (0.00–13.06), *p* = 0.10
Positive affects	*F* = 7.41 (0.52–22.18), *p* = 0.008, 6.28
Arthritis severity	*F* = 1.38 (0.00–6.09), *p* = 0.24
Age	*F* = 0.28 (0.00–6.09), *p* = 0.59
Gender	*F* = 1.15 (0.00–9.22), *p* = 0.28

Arthritis severity was based on severity of bone and joint damage on radiographs

* Effect size estimates were based on noncentrality parameter for the *F* test and were provided for significant *p* values.

Age had an independent association with the scores of fear-avoidance thoughts and positive and negative affect domains, in relation to perceived walking ability (Table 2). More specifically, older patients with less fear of injury/catastrophizing thoughts and a better positive affect overestimated their actual walking speed. Gender had an independent association with fear avoidance and positive affect with men having less fear avoidance and higher positive affect (e.g. higher coping abilities and better pain resilience). There was a reverse association between severity of arthritis and discordance. In other words, more arthritic changes on imaging was associated with less discordance (Table 2).

## Discussion

The results of this study further demonstrate the importance of using a biopsychosocial approach in the assessment and management of chronic pain secondary to arthritis, detailing key psychological factors that impact patient perception of pain and function. In the context of age-related physical disability, often related to degenerative joint disease, two key explanatory factors were identified in this study. First, patients with less fear avoidance who positively confront the disabling activity-related pain and fear of activity perceive their function to be better. Secondly, positive affect was more critical than negative affect in the OA population. Positive affect is associated with a positive outlook, higher resilience, more optimism, and better coping abilities and is highly associated with less perceived disability. Negative affect, characterized by depressive symptoms, higher anxiety, and overreaction to minimal stressors is still related to discordance and perceived disability but has a less critical role than positive affect. Plys and Desrichard (2020) found that individuals with higher positive affect perceived their goals as less difficult, more attainable, and more congruent with their self-identity and individuals with higher negative affect were less likely to adhere to their goals, as they perceive the tasks as more difficult, more stressful, less attainable, and less controllable. The overestimation of physical capability appears to be related to a well-established psychological phenomenon, the response shift, which uses recalibration of internal standards to support a more stable and fulfilling quality of life (Daltroy et al., 1999; Razmjou et al., 2006, 2009; Schwartz and Sprangers, 1999).

### Role of psychosocial factors in perceived and actual functional discordance

The discrepancy between self-report and performance-based measures has been well-studied in elderly population (Feuering et al., 2014; Guralnik et al., 1989; Henstra et al., 2022; Kempen et al., 1996; Kok et al., 2020; Merrill et al., 1997; Wittink et al., 2003; Wloch, 2016). The topic is less addressed in the OA literature, with a limited number of studies examining the impact of anxiety, depression, age, and gender on this concept (Dayton et al., 2016; Lentz et al., 2020; Pua et al., 2023; Twardzik et al., 2024; Wilfong et al., 2020). According to adaptation capability, which explains one component of “response shift theory,” shifting internal standards by recalibrating self-assessment helps to better cope with health-related challenges and normally occurring age-related restrictions. Response shift is a psychological construct, whereby changing internal standards, values, or reconceptualization of health-related quality of life helps to better cope with negative health-related experiences (Schwartz and Sprangers, 1999). In fact, response shift is directly related to the person’s appraisal change. In one of the early publications about response shift in the field of orthopedics, response shift was indeed responsible for change of internal standards in patients who underwent total knee replacement (Razmjou et al., 2006, 2009). A recent study that has used a clinical prediction model in patients with hip arthroplasty has also revealed that the most important predictive factors of post-surgical outcome were cognitive appraisal processes, further demonstrating the importance of mental well-being in outcome-based research (Sniderman et al., 2021). Response shift and cognitive appraisal are significant contributors to outcome-based research and are relevant to arthroplasty populations and need to be incorporated into routine clinical assessment (Ong et al., 2010; Razmjou et al., 2006, 2009; Schwartz et al., 2022; Sniderman et al, 2021).

In terms of other aspects of mental well-being, studies have shown that underestimation of daily functioning is linked to factors such as depressive or anxiety symptoms and a low sense of control (Daltroy et al., 1999; Henstra et al., 2022; Kempen et al., 1996; Wittink et al., 2003). Henstra et al. (2022) reported that having a negative affect was associated with underestimation of function. The underestimation may lead to substantial underutilization of one’s physical capacity and increased dependence (Kempen et al., 1996). It is likely that underestimating physical function is related to negative dispositional traits (e.g. pain focused and catastrophic behaviors).

One of the interesting findings of the present study was the more important role of having a positive affect versus not having a negative affect in perceived functional abilities. The positive affect domain of OSPRO-YF is derived from scales that examine self-efficacy for pain and rehabilitation, and the acceptance for chronic pain. The negative mood domain of OSPRO-YF is derived from scales that address the degree of depressive symptoms, dispositional anxiety, and anger symptoms. Having a positive outlook comes from having higher resilience, a positive interpretation of negative experiences, and better coping abilities, ultimately leading to more independence and a better quality of life. Role of positive coping cognition is noted to be related to resilience to pain, which is characterized by psychological flexibility and resourcefulness to adaptively cope with psychological distress (Lentz et al., 2020; Ong et al., 2010). The strong focus on behavioral aspects of pain coping in the positive affect domain appears to be more important to the OA population than not having general symptoms of depression, anger, and anxiety. Different aspects of mental well-being could be strengthened through cognitive behavioral therapy (CBT) in patients with arthritis and chronic pain.

In the present study, fear-avoidance was the most significant contributor to discordance in relation to both walking and sitting activities. It is likely that a fear-initiated avoidant behavior is responsible for overestimating the occurrence or exacerbation of pain during simple daily activities. The exaggerated negative orientation toward pain experiences will ultimately result in more distress, chronic pain, physical deconditioning, depression, and increased healthcare utilization and costs (e.g. unnecessary surgical management). Similarly, confronting the fear of activity-related pain will help to minimize the impact of age-related decline and loss of independence.

### Role of patient characteristics in the pattern of discordance

In the present study, age showed a differential pattern of relationship with the amount of discordance. The older individuals tended to show less discordance than younger patients. This may indicate that older patients with OA had a more realistic perception of their function. Younger patients have higher expectations with respect to daily activities, which may be reflected by reporting worse function compared to performance-based function. Similar to our study, Wilfong et al. (2020) reported greater concordance between the self-report and performance-based measures with increasing age. Henstra et al. (2022) reported that older individuals tended to overestimate their level of physical functioning and the extent of overestimation increased with time. Similarly, Lentz et al. (2020) reported that patients with a higher distress had the lowest mean age. Thus, overestimation may partly reflect normal aging rather than a pathological process. Objective measures of physical functioning may be capable of detecting a small functional decline which may be unnoticeable to an aging person (Guralnik et al., 1989; Kok et al., 2020). Older adults in general appear to benefit from recalibration of their perceived function, which may help to maintain equilibrium in quality of life, despite having more difficulty executing specific tasks or require assistive devices (Daltroy et al., 1999). The exact direction of recalibration in relation to age needs more examination in larger samples of patients with OA.

In the present study, gender was associated with discordance of self-report/walking ability only when positive affect and fear avoidance behavior were taken into account. Role of gender in accounting for discrepancy between self-report and performance-based measures has been discussed in the literature. Wilfong et al. (2020) have highlighted the combined role of gender and obesity in discordance between self-reported function compared to the performance-based function. In elderly population, male gender has been associated with overestimation of capacity of function on self-report outcomes (Kok et al., 2020). Conversely, female gender has been associated with underestimation of function (Daltroy et al., 1999; Elboim-Gabyzon et al., 2012; Fors et al., 2006; Kempen et al., 1996; Kok et al., 2020; Merrill et al., 1997; Wloch, 2016). In other words, men appear to see themselves in a more optimistic light than their female counterpart when it comes to perception of their physical capabilities. Severity of arthritis showed a negative association with discordance, in that, more pathological changes on imaging was associated with less discordance. This may indicate that patients with less degenerative changes may show a more complex pattern of adaptation.

In summary, self-report and performance-based outcome measures provide distinct and complimentary information about the functional abilities of a person with OA. By incorporating outcome measures that assess the psychosocial element of well-being and cognitive appraisal, we will close the gap and will have a clearer picture of challenges that a person with arthritis faces. The present study showed that positive affect had a more superior role in perception of disability. Positive affect is characterized by resilience, joy, and high levels of energy, concentration, enthusiasm, and alertness, whereas negative affect is characterized by distress, anger, contempt, nervousness, or fear (Watson et al., 1988). A clear influence of dispositional optimism on the recovery has been established after joint arthroplasty (Balck et al., 2016). The positive and negative traits and mental states are expected to affect the way individuals perceive their function and attainability of their rehabilitation goals (Plys and Desrichard, 2020).

The clinical implication of our findings for joint arthroplasty candidates is emphasizing the role of psychosocial factors as potential barriers to recovery and recognizing the value of adjunct psychological interventions to overcome these barriers. By considering the impact of psychological, social, and personality characteristics on disability and recovery, additional treatment options can be introduced in the management of OA. For example, emotional regulation strategies and cognitive behavioral therapy could be applied to reinforce independence by reducing fear-avoidant behavior and developing better coping techniques, higher resilience, and a more positive outlook, while navigating a chronic and disabling condition.

## Conclusion

This study confirmed that fear avoidance beliefs and positive affect play important roles in perception of pain and function, as shown through lack of concordance between self-report and performance-based outcomes. Age, gender, and severity of arthritis are contributing factors to the existing discrepancy and should be taken into consideration for a more holistic approach to arthritis management.

## References

[bibr1-13591053241253895] Balck F Lippmann M Jeszenszky C , et al. (2016) The influence of optimism on functionality after total hip replacement surgery. Journal of Health Psychology 21(8): 1758–1767. 25609405 10.1177/1359105314566256

[bibr2-13591053241253895] Binkley JM Stratford PW Lott SA , et al. (1999) The lower extremity functional scale (LEFS): Scale development, measurement properties, and clinical application. North American orthopaedic rehabilitation research network. Physical Therapy 79(4): 371–383. 10201543

[bibr3-13591053241253895] Chen X Tang H Lin J , et al. (2023) Temporal trends in the disease burden of osteoarthritis from 1990 to 2019, and projections until 2030. PLoS One 18(7): e0288561. 10.1371/journal.pone.0288561 PMC10365297 37486949

[bibr4-13591053241253895] Coman L Richardson J (2006) Relationship between self-report and performance measures of function: A systematic review. Canadian Journal on Aging 25(3): 253–270. 17001588 10.1353/cja.2007.0001

[bibr5-13591053241253895] Daltroy LH Larson MG Eaton HM , et al. (1999) Discrepancies between self-reported and observed physical function in the elderly: The influence of response shift and other factors. Social Science & Medicine 48(11): 1549–1561. 10400256 10.1016/s0277-9536(99)00048-9

[bibr6-13591053241253895] Dayton MR Judd DL Hogan CA , et al. (2016) Performance-based versus self-reported outcomes using the hip disability and osteoarthritis outcome score after total hip arthroplasty. American Journal of Physical Medicine & Rehabilitation 95(2): 132–138. 26259051 10.1097/PHM.0000000000000357 PMC4772958

[bibr7-13591053241253895] Dobson F Hinman RS Roos EM , et al. (2013) OARSI recommended performance-based tests to assess physical function in people diagnosed with hip or knee osteoarthritis. Osteoarthritis and Cartilage 2013; 21(8): 1042–1052. 23680877 10.1016/j.joca.2013.05.002

[bibr8-13591053241253895] Elboim-Gabyzon M Rozen N Laufer Y (2012) Gender differences in pain perception and functional ability in subjects with knee osteoarthritis. International Scholarly Research Notices 2012: 413105. 10.5402/2012/413105 PMC4063163 24977076

[bibr9-13591053241253895] Engel GL (1977) The need for a new medical model: A challenge for biomedicine. Science 196(4286): 129–136. 847460 10.1126/science.847460

[bibr10-13591053241253895] Epstein RM (2014) Realizing Engel’s biopsychosocial vision: Resilience, compassion, and quality of care. The International Journal of Psychiatry in Medicine 47(4): 275–287. 25084850 10.2190/PM.47.4.b

[bibr11-13591053241253895] Feuering R Vered E Kushnir T , et al. (2014) Differences between self-reported and observed physical functioning in independent older adults. Disability and Rehabilitation 36(17): 1395–1401. 24001263 10.3109/09638288.2013.828786

[bibr12-13591053241253895] Fors S Thorslund M Parker MG (2006) Do actions speak louder than words? Self-assessed and performance-based measures of physical and visual function among old people. European Journal of Ageing 22; 3(1): 15–21. 10.1007/s10433-006-0021-5 PMC5546355 28794746

[bibr13-13591053241253895] George SZ Beneciuk JM Lentz TA , et al. (2017) The optimal screening for prediction of referral and outcome (OSPRO) in patients with musculoskeletal pain conditions: A longitudinal validation cohort from the USA. BMJ Open 7(6): e015188. 10.1136/bmjopen-2016-015188 PMC5734477 28600371

[bibr14-13591053241253895] George SZ Li C Luo S , et al. (2020) Longitudinal monitoring of pain associated distress with the optimal screening for prediction of referral and outcome yellow flag tool: Predicting reduction in pain intensity and disability. Archives of Physical Medicine and Rehabilitation 101(10): 1763–1770. 32599059 10.1016/j.apmr.2020.05.025

[bibr15-13591053241253895] Grinker RRS (1994) Training of a psychiatrist-psychoanalyst. Journal of the American Academy of Psychoanalysis 22(2): 343–350. 7961048 10.1521/jaap.1.1994.22.2.343

[bibr16-13591053241253895] Guralnik JM Branch LG Cummings SR , et al. (1989) Physical performance measures in aging research. Journal of Gerontology 44(5): 141. 10.1093/geronj/44.5.m141 2768767

[bibr17-13591053241253895] Henstra M Rhebergen D van der Velde N , et al. (2022) Patterns of discordance of physical functioning in older persons; different associations for apathy and depression? Results from the NESDO-study. Aging & Mental Health 26(8): 1580–1588. 34124966 10.1080/13607863.2021.1932738

[bibr18-13591053241253895] Jones CJ Rikli RE Beam WC (1999) A 30-s chair-stand test as a measure of lower body strength in community-residing older adults. Research Quarterly for Exercise and Sport 70(2): 113–119. 10380242 10.1080/02701367.1999.10608028

[bibr19-13591053241253895] Kempen GI van Heuvelen MJ van den Brink RH , et al. (1996) Factors affecting contrasting results between self-reported and performance-based levels of physical limitation. Age Ageing 25(6): 458–464. 9003883 10.1093/ageing/25.6.458

[bibr20-13591053241253895] Kennedy DM Stratford PW Riddle DL , et al. (2008) Assessing recovery and establishing prognosis following total knee arthroplasty. Physical Therapy 88(1): 22–32. 17986495 10.2522/ptj.20070051

[bibr21-13591053241253895] Kok AAL Henstra MJ van der Velde N , et al. (2020) Psychosocial and health-related factors associated with discordance between 13-year trajectories of self-reported functional limitations. Journal of Aging and Health 32(9): 1084–1097. 31686583 10.1177/0898264319884404 PMC7731649

[bibr22-13591053241253895] Lentz TA Beneciuk JM Bialosky JE , et al. (2016) Development of a yellow flag assessment tool for orthopaedic physical therapists: Results from the optimal screening for prediction of referral and outcome (OSPRO) cohort. Journal of Orthopaedic & Sports Physical Therapy 46(5): 327–343. 26999408 10.2519/jospt.2016.6487

[bibr23-13591053241253895] Lentz TA George SZ Manickas-Hill O , et al. (2020) What general and pain-associated psychological distress phenotypes exist among patients with hip and knee osteoarthritis? Clinical Orthopaedics and Related Research^®^ 478(12): 2768–2783. 33044310 10.1097/CORR.0000000000001520 PMC7899410

[bibr24-13591053241253895] Master H Coleman G Dobson F , et al. (2021) A narrative review on measurement properties of fixed-distance walk tests up to 40 meters for adults with knee osteoarthritis. The Journal of Rheumatology 48(5): 638–647. 33060316 10.3899/jrheum.200771

[bibr25-13591053241253895] Mehta SP Fulton A Quach C , et al. (2016) Measurement properties of the lower extremity functional scale: A systematic review. Journal of Orthopaedic & Sports Physical Therapy 46(3): 200–216. 26813750 10.2519/jospt.2016.6165

[bibr26-13591053241253895] Merrill SS Seeman TE Kasl SV , et al. (1997) Gender differences in the comparison of self-reported disability and performance measures. The Journals of Gerontology Series A: Biological Sciences and Medical Sciences 52(1): 19. 10.1093/gerona/52a.1.m19 9008665

[bibr27-13591053241253895] Myers AM Holliday PJ Harvey KA , et al. (1993) Functional performance measures: Are they superior to self-assessments? Journal of Gerontology 48(5): 196. 10.1093/geronj/48.5.m196 8366262

[bibr28-13591053241253895] Ong AD Zautra AJ Reid MC (2010) Psychological resilience predicts decreases in pain catastrophizing through positive emotions. Psychology and Aging 25(3): 516–523. 20853962 10.1037/a0019384 PMC3626095

[bibr29-13591053241253895] Papadimitriou G (2017) The “biopsychosocial model”: 40 years of application in psychiatry. Psychiatriki 28(2): 107–110. 28686557 10.22365/jpsych.2017.282.107

[bibr30-13591053241253895] Plys E Desrichard O (2020) Associations between positive and negative affect and the way people perceive their health goals. Frontiers in Psychology 11: 334. 32194483 10.3389/fpsyg.2020.00334 PMC7063053

[bibr31-13591053241253895] Pua Y Tan BY Low J , et al. (2023) Discordance between self-reported and performance-based physical function in patients who have knee osteoarthritis: Associations with pain intensity and negative affect. The Journal of Arthroplasty 38: 1705–1713. 36940758 10.1016/j.arth.2023.03.044

[bibr32-13591053241253895] Razmjou H Palinkas V Robarts S , et al. (2021) Psychometric properties of the OSPRO-YF screening tool in patients with shoulder pathology. Physiotherapy Canada 73(1): 26–36. 35110821 10.3138/ptc-2019-0046 PMC8774952

[bibr33-13591053241253895] Razmjou H Schwartz CE Yee A , et al. (2009) Traditional assessment of health outcome following total knee arthroplasty was confounded by response shift phenomenon. Journal of Clinical Epidemiology 62(1): 91–96. 19095168 10.1016/j.jclinepi.2008.08.004

[bibr34-13591053241253895] Razmjou H Yee A Ford M , et al. (2006) Response shift in outcome assessment in patients undergoing total knee arthroplasty. The Journal of Bone & Joint Surgery 88(12): 2590–2595. 17142408 10.2106/JBJS.F.00283

[bibr35-13591053241253895] Reuben DB Seeman TE Keeler E , et al. (2004) Refining the categorization of physical functional status: The added value of combining self-reported and performance-based measures. The Journals of Gerontology Series A: Biological Sciences and Medical Sciences 59(10): 1056–1061. 15528778 10.1093/gerona/59.10.m1056

[bibr36-13591053241253895] Robarts S Denis S Kennedy D , et al. (2021) Patient gender does not influence referral to an orthopaedic surgeon by advanced practice orthopaedic providers: A prospective observational study in Canada. BMC Health Services Research 21(1): 952–955. 34511124 10.1186/s12913-021-06965-5 PMC8435171

[bibr37-13591053241253895] Robarts S Palinkas V Boljanovic D , et al. (2023a) Validity of the virtual severity scoring system in patients with knee osteoarthritis. Orthopaedic Proceedings 105(SUPP_8): 51.

[bibr38-13591053241253895] Robarts S Razmjou H Yee A , et al. (2023b) Risk stratification in a tertiary care spine centre: Comparison between STarTBack and OSPRO-YF screening tools. Physiotherapy Canada 75(2): 158–166. 37736380 10.3138/ptc-2021-0026 PMC10510560

[bibr39-13591053241253895] Schwartz CE Rapkin BD Sniderman J , et al. (2022) Appraisal and patient-reported outcomes following total hip arthroplasty: A longitudinal cohort study. Journal of Patient-Reported Outcomes 6(1): 93. 36064834 10.1186/s41687-022-00498-z PMC9445109

[bibr40-13591053241253895] Schwartz CE Sprangers MA (1999) Methodological approaches for assessing response shift in longitudinal health-related quality-of-life research. Social Science Medicine 48(11): 1531–1548. 10400255 10.1016/s0277-9536(99)00047-7

[bibr41-13591053241253895] Sniderman J Stark RB Schwartz CE , et al. (2021) Patient factors that matter in predicting hip arthroplasty outcomes: A machine-learning approach. The Journal of Arthroplasty 36(6): 2024–2032. 33558044 10.1016/j.arth.2020.12.038

[bibr42-13591053241253895] Spadoni GF Stratford PW Solomon PE , et al. (2004) The evaluation of change in pain intensity: A comparison of the P4 and single-item numeric pain rating scales. Journal of Orthopaedic & Sports Physical Therapy 34(4): 187–193. 15128188 10.2519/jospt.2004.34.4.187

[bibr43-13591053241253895] Swain S Coupland C Sarmanova A , et al. (2023) Healthcare utilization and mortality in people with osteoarthritis in the UK: Findings from a national primary care database. British Journal of General Practice 73(733): e615–e622. 10.3399/BJGP.2022.0419 PMC10355815 37429733

[bibr44-13591053241253895] Twardzik E Schrack JA Freedman VA , et al. (2024) An incomplete model of disability: Discrepancies between performance-based and self-reported measures of functioning. The Journals of Gerontology, Series A: Biological Sciences and Medical Sciences 79(4): glad 271. 10.1093/gerona/glad271 PMC10959443 38071606

[bibr45-13591053241253895] Watson D Clark LA Tellegen A (1988) Development and validation of brief measures of positive and negative affect: The PANAS scales. Journal of Personality and Social Psychology 54: 1063–1070. 3397865 10.1037//0022-3514.54.6.1063

[bibr46-13591053241253895] Wilfong JM Badley EM Power JD , et al. (2020) Discordance between self-reported and performance-based function among knee osteoarthritis surgical patients: Variations by sex and obesity. PLoS One 15(7): e0236865. 10.1371/journal.pone.0236865 PMC7392249 32730319

[bibr47-13591053241253895] Wittink H Rogers W Sukiennik A , et al. (2003) Physical functioning: Self-report and performance measures are related but distinct. Spine 28(20): 2407–2413. 14560092 10.1097/01.BRS.0000085304.01483.17

[bibr48-13591053241253895] Wloch EG (2016) Exploring the discordance between self-reported and performance-based measures of physical capability. PhD Thesis, UCL (University College London), UK.

